# Training evaluation: a case study of training Iranian health managers

**DOI:** 10.1186/1478-4491-7-20

**Published:** 2009-03-05

**Authors:** Maye Omar, Nancy Gerein, Ehsanullah Tarin, Christopher Butcher, Stephen Pearson, Gholamreza Heidari

**Affiliations:** 1 Nuffield Centre for International Health and Development, University of Leeds, Leeds, UK; 2 World Health Organization, Khartoum, Sudan; 3 Staff and Departmental Development Unit, University of Leeds, Leeds, UK; 4 Ministry of Health and Medical education, Tehran, Islamic Republic of Iran

## Abstract

**Background:**

The Ministry of Health and Medical Education in the Islamic Republic of Iran has undertaken a reform of its health system, in which-lower level managers are given new roles and responsibilities in a decentralized system. To support these efforts, a United Kingdom-based university was contracted by the World Health Organization to design a series of courses for health managers and trainers. This process was also intended to develop the capacity of the National Public Health Management Centre in Tabriz, Iran, to enable it to organize relevant short courses in health management on a continuing basis. A total of seven short training courses were implemented, three in the United Kingdom and four in Tabriz, with 35 participants. A detailed evaluation of the courses was undertaken to guide future development of the training programmes.

**Methods:**

The Kirkpatrick framework for evaluation of training was used to measure participants' reactions, learning, application to the job, and to a lesser extent, organizational impact. Particular emphasis was put on application of learning to the participants' job. A structured questionnaire was administered to 23 participants, out of 35, between one and 13 months after they had attended the courses. Respondents, like the training course participants, were predominantly from provincial universities, with both health system and academic responsibilities. Interviews with key informants and ex-trainees provided supplemental information, especially on organizational impact.

**Results:**

Participants' preferred interactive methods for learning about health planning and management. They found the course content to be relevant, but with an overemphasis on theory compared to practical, locally-specific information. In terms of application of learning to their jobs, participants found specific information and skills to be most useful, such as health systems research and group work/problem solving. The least useful areas were those that dealt with training and leadership. Participants reported little difficulty in applying learning deemed "useful", and had applied it often. In general, a learning area was used less when it was found difficult to apply, with a few exceptions, such as problem-solving. Four fifths of respondents claimed they could perform their jobs better because of new skills and more in-depth understanding of health systems, and one third had been asked to train their colleagues, indicating a potential for impact on their organization. Interviews with key informants indicated that job performance of trainees had improved.

**Conclusion:**

The health management training programmes in Iran, and the external university involved in capacity building, benefited from following basic principles of good training practice, which incorporated needs assessment, selection of participants and definition of appropriate learning outcomes, course content and methods, along with focused evaluation. Contracts for external assistance should include specific mention of capacity building, and allow for the collaborative development of courses and of evaluation plans, in order to build capacity of local partners throughout the training cycle. This would also help to develop training content that uses material from local health management situations to demonstrate key theories and develop locally required skills. Training evaluations should as a minimum assess participants' reactions and learning for every course. Communication of evaluation results should be designed to ensure that data informs training activities, as well as the health and human resources managers who are investing in the development of their staff.

## Background

"Capacity" is a frequently-used term in development discourse, defined as the "ability of people, organizations and society as a whole to manage their affairs successfully" [1]. The three levels referred to – individual, organizations and society – are closely connected and interdependent in terms of using capacities. At the individual level, capacities focus on the skills and knowledge of people. Organizations provide a framework for individuals' capacities to connect and achieve collective goals, through providing facilities such as information technology equipment, access to journals and funds. Larger systems (society) provide an enabling environment in which the organizations can function, such as the overall policies, rules, norms and values governing their mandates and modes of operation [2].

This paper focuses on the level of individual capacity – the knowledge, skills and confidence that people have to make effective use of their abilities – and the role of training in developing them, by analysing the results of an evaluation of a training programme for Iranian health professionals. It goes on to consider the implications for future training and evaluation efforts.

The health workforce is made up of two overall groups: health service providers, and health management and support workers. The latter, as the *World health report 2006 *[3] discusses, constitute the "invisible backbone of the health system"; any shortage in terms of their number or skills would adversely affect the performance of the system. While in some countries the two groups are distinct entities, in others they often perform both functions. Interestingly, in the context of Iran, health workers serve as service providers as well as health service managers and academics, as implied by the name: Ministry of Health and Medical Education.

There is a widespread shortage of health management cadres, requiring urgent attention to increase the amount, diversity and quality of training [3]. Their training needs to be continuously updated, adapting to new contexts and needs, and to be evaluated in order to know whether training methods have been effective and if identified needs have been met. Training inputs can represent a significant investment for an organization; both human resources and service managers need to decide whether training generates value proportional to the investment in terms of improved job performance and organizational outcomes, given the competition for scarce resources in organizations and the need to be accountable for financial decisions. Even though enormous investments have been and continue to be made in capacity development of the health sector in low-income countries, including in training programmes, there are few published evaluations of such training programmes for health professionals.

The training evaluated in this study was designed to achieve two main outcomes: first, to develop the competences of participants in their current management roles and responsibilities in order to enable them to do their jobs better, and second, to enable participants to organize and manage the training of others using a range of methods and approaches, i.e. to train the future trainers.

### Evaluation of training: conceptual framework

As defined by Birchall and Smith [4], training encompasses the systematic preparation of individuals to develop their capacity to perform functions valued socially and by the marketplace. It comprises the full continuum of education, skill formation processes and training activities, and is one of the pillars on which an integrated human resource development strategy must be based.

The training process should start with the assessment of the skills and knowledge needed to achieve organizational objectives, and a consideration of whether training is the most appropriate solution to meeting the knowledge and skill needs. If training is appropriate, the selection of the most suitable participants is the next essential stage in the process. Having clarified organizational purpose, suitability and audience, the objectives of the training programme can be developed and the most appropriate way to evaluate training outcomes can be selected.

The most widely used framework for evaluation is that of Kirkpatrick [5]. The four levels in this framework are: participant reactions, learning, behaviour depicted as application of knowledge, and organizational changes. Phillips and Phillips [6] add one level to this, to include return on investment, rather like cost-benefit analysis, as shown in Table 1.

**Table 1:** Levels of training programme evaluation

**Level**	**What is measured**
1. Reaction	Participants' reaction to the training programme and stakeholder satisfaction with it

2. Learning	Knowledge, skills or attitude changes of participants, related to the training programme

3. Application	Also called Training Transfer: use of new knowledge and skills back on the job

4. Impact	Changes in the organization related to the programme

5. Return on investment	Monetary value of the impact compared to the costs of the training programme

Not all training programmes should be evaluated at all levels: the possible significance of information gained must be assessed against the costs and time of obtaining it. Probably all programmes should be evaluated at level 1, and most at level 2. Level 3, application, is of particular interest to trainees' organizations. Gathering information on impact and return on investment is more difficult, complex and costly.

The issue of measuring the impact of training is well represented in the literature, as there are attempts in both secondary and tertiary education to gain evidence of training efficacy. Flecknoe [7] notes the requirement of United Kingdom Teacher Training Agency-funded courses for schoolteachers to demonstrate impact on pupils, and reports that the attempts to measure impact are not working and are seen as "inconsistent, lacking validity and reliability ... imposing excessive burdens, insufficiently promoting quality enhancement, and representing poor value for money" [8].

One large study of teachers' professional development courses found that participants' reactions were usually or always assessed in 75% of courses, and participant learning in 40% of courses, but application and organizational change were assessed usually or always only 30% to 40% of the time [9]. Flecknoe [7] concludes that the question is not whether providers of continuing professional development should be accountable for impact, but rather whether it is reasonable and feasible to assess it: is the lack of evidence because of the lack of impact, or because it is too difficult to measure?

Prebble et al. [10], in their review of 150 studies worldwide, considered all formats of development interventions: short courses; development within peer groups/peer review; and intensive (teacher training) programmes. They found little evidence of the long-term efficacy of short training courses [11,12]. One survey suggested that only 50% of training investments eventually yield individual organizational improvements [13]. However, short training courses were noted as most effective for dissemination of information and training in discrete skills and techniques [10]. Most reported evaluations of short courses are based on the immediate views of participants. Notable exceptions are Rust [14] and Brew and Lublin [15]: both studies reported, based on follow-up interviews, high proportions of staff claiming to have applied the ideas gained in the short courses.

### The training programme

The Ministry of Health and Medical Education of Iran has been making significant but sporadic efforts at reforming the health system, but a real impetus came with the publication of the *2000 World health report: Health systems: improving performance *[16]. The full text was translated into Farsi – the local language – and subsequently, at the request of the Ministry of Health and Medical Education, the World Health Organization (WHO) allocated 25% of its programme budget (2002–2003) for Iran to supporting health sector reforms. A project was designed with four components: (1) defining a universal minimum basic health services package and strengthening the referral system; (2) assuring stewardship and good governance; (3) improving planning and management, including structural changes such as decentralization; and (4) improving the health financing and payment mechanisms [17]. The World Bank agreed to fund the project.

Training is particularly important during health sector reforms that involve forms of decentralization, as lower-level managers are given new roles and responsibilities [18,19]. One of the activities planned as a part of the WHO programme was to carry out an assessment of training needs, to support the reforms being discussed. This activity, undertaken in November 2003, recommended: (1) sending candidates to foreign institutes for training in selected areas, and (2) developing the capacity of a local institute to organize relevant short courses on a continuing basis [20].

Following the needs assessment, WHO implemented a project to train health staff and build the capacity of a National Public Health Management Centre, at Tabriz University of Medical Sciences, enabling it to organize short courses on health planning and management for middle-level health managers [21]. The National Public Health Management Centre (NPMC) was established as a national centre for training and research in health planning and management, but until this time there had been no organized effort for the in-service training of health managers.

Accordingly, 19 Iranian health officials were sent by the Ministry of Health and Medical Education on three courses at the Nuffield Centre for International Health and Development (Nuffield Centre), University of Leeds, United Kingdom, in 2005 (Table 2). Also, the Nuffield Centre designed and conducted four short courses at the NPMC, Tabriz, in 2005. A cohort of 22 health officials was selected by the Ministry of Health and Medical Education to participate in the NPMC courses, although the numbers participating varied by course. In total, 35 individuals participated in the short courses at the Nuffield Centre or NPMC (Table 2).

**Table 2:** Details of courses

**Title**	**Location**	**Date (duration)**	**Summary of content**	**Number of participants**
Health system decentralization	NCIHD Leeds, UK	January 2005 (5 weeks)	Health policy & planning; health economics; public health interventions; effective decentralization	8

Clinical governance	NCIHD Leeds, UK	February 2005 (5 weeks)	Health systems development; quality improvement; planning cycle; measuring performance	5

Health planning, management and policy	NCIHD Leeds, UK	September – December 2005 (10 weeks)	Health Management, Planning and Policy	6

Policy context for health sector reform	NPMC Tabriz, Iran	May 2005 (1 week)	Health sector reforms; equity; Iranian health policy context; health financing; priority setting	21

Planning and organization of health sector reform	NPMC Tabriz, Iran	July 2005 (1 week)	Information for planning; strategic and leadership skills; communication skills; team work; problem analysis; project management	18

Resources management for health sector reform	NPMC Tabriz, Iran	August 2005 (1 week)	Resources; capacity strengthening; quality assessment; monitoring and evaluation; stakeholder involvement; dissemination	21

Training of trainers (TOT)	NPMC Tabriz, Iran	October 2005 (1 week)	Identifying training needs; learning outcomes; effective presentations; small learning groups; training course practicalities; evaluation	30

Principles of adult learning were followed throughout the training. These involved the use of a number of interactive tools and techniques suitable for training adults, as well as intramodular assignments based on management issues in the participants' organizations, which were discussed in the succeeding module. Training techniques aimed to use the experience of trainees as the basis for new learning. Examples included trainees' applying their experience to tasks in group exercises, and working on case studies and projects.

A staged approach with continuous learning was used for the following reasons:

• It offered a practical solution to the difficulty staff find in taking time away from routine activities to attend training courses.

• It allowed for reflective learning. Reflection is a process of digesting new information or experience; it helps to understand and then apply learning to structured tasks.

• It facilitated use of self-development activities, such as personal plans and projects. These types of learning tools allowed for reflection and personalized learning, making it more relevant and interesting.

These activities took place over the course of the training period with support from training mentors and line managers.

This paper discusses the findings of an evaluation of the training conducted in Leeds and Tabriz designed to build the capacity of Iranian management and training staff. The paper reports on achievement against the four levels of the Kirkpatrick model. The evaluation methodology is also discussed, and recommendations made for future efforts in capacity building.

## Methods: questionnaire and interviews

A questionnaire was used to collect quantitative and qualitative information from a sample of course participants. It was based, with the kind permission of its main author, on one used in a study to evaluate the "Effects of Postgraduate Certificates in Teaching and Learning in Higher Education" [22]. The questionnaire was developed in Leeds, discussed and adapted with NPMC staff, and pilot-tested.

The questionnaire was designed to elucidate reaction, learning and application of learning. The five sections were:

1. background information on the respondents, including any change in job role;

2. the importance of different methods for their learning about health planning and management;

3. perceptions of the overall course – content, organization, value;

4. perceptions of the usefulness of the course material and its application;

5. transfer of knowledge from the courses to do their current job.

In total, 23 of the 35 training participants (66%) completed the questionnaire in September 2006 (Table 3). The remainder could not be contacted, or declined. While this group was self-selected, in that responding was voluntary, Table 3 shows that the data collected generally are representative of all training participants. Questionnaire data were entered and cleaned using SPSS software. Descriptive and cross-tabulations were generated using SPSS.

**Table 3:** Number of training participants and questionnaire respondents

**Site of course**		**Time between end of last course and completing questionnaire**	**Number of participants**	**Number completing questionnaire**	**%**
				
NCIHD, Leeds	NPHC, Tabriz				
✓		12 months	13	7	54

	✓	1–3 months	16	10	63

✓	✓	1–3 months	6	6	100

		Total	35	23	66

In-depth interviews were held with key individuals involved in the project: two with NPMC managers of the training courses, two with national managers of the health sector reform project, and one with the WHO Iran office. A group interview was also held with five trainees from the courses in Nuffield Centre. The purpose of the interviews was to gain insights into the application and impact of the training, enrich the findings from the questionnaire and clarify uncertainties.

All in-depth and group interviews took place in Tehran and were conducted in English. As there were few interviews, the researcher decided to take note of the discussions and transcribe near verbatim. This method made the discussion less formal and enabled respondents to feel relaxed and to talk openly. A detailed summary sheet for each discussion was used as a data organizational tool. Data were analysed using common themes derived from the question guide and quotations are used to illustrate some of the findings.

## Results

### General characteristics of participants

The respondents were predominantly male, early middle-aged, health personnel with backgrounds in planning and management, most of whom worked at provincial universities and therefore had both health system and academic responsibilities (Table 4). When asked the reasons for their selection for the course, the majority said it was to improve their organization's performance (83%), to enable them to take up new functions (35%) and to get a promotion (22%). None of the candidates saw the opportunity of attending courses in Leeds as a reward by their superiors.

**Table 4:** Characteristics of respondents

**Indicator**	**% *(n = 23)***
**Sex**	
Male	65.0
Female	35.0

**Age**	
21–29	8.7
30–39	56.5
40–49	17.4
Over 49	17.4

**Main area of work**	
Policy, planning and management	73.9
Health care provision	30.4
Academic	13.0
Other	13.0

**Years in current job**	
< 2	13.0
2–4	39.1
5–9	26.1
> 9	21.7

**Place of work**	
National level	43.5
Provincial university	73.9
District level	4.3

A key consideration in effective training is the appropriate selection of candidates. Training is most effective for people who have the required intellectual ability, feel the training is useful to help them perform better and will benefit their career, and work in a supportive organization [13]. In this project, the selection of participants was limited by the participants' English language skills. As a key informant noted, "not the most appropriate students were selected", meaning that sometimes people from some areas particularly in need of capacity building, or those from pilot reform project areas, were not able to attend. Similarly, a decision-maker reported that selection was due to a person's having "a reasonable knowledge of English", albeit, he noted, "they might not have been the right candidates".

At the time of the evaluation, all except one of the respondents were in the same post as before their training, although 11 of the 23 reported getting involved in new functions: three noted that they were now acting as trainers, and eight reported taking on other functions, such as management of health facilities in the provinces. Eight respondents viewed the training as having helped them to perform their job better; four of this group were trainers.

That 11 of 23 respondents reported taking on new functions may be considered a good result, given that 10 of the respondents were questioned one to three months after their training, and thus had only a brief opportunity to try out new skills. These observations were amplified in a group interview with the trainees, who were unanimous in the view that at least some participants were in the right position to initiate change, but weaknesses in the support system (in the Ministry of Health and Medical Education) prevented them from applying what they had learnt.

### Learning for health planning and management

**Figure 1:**
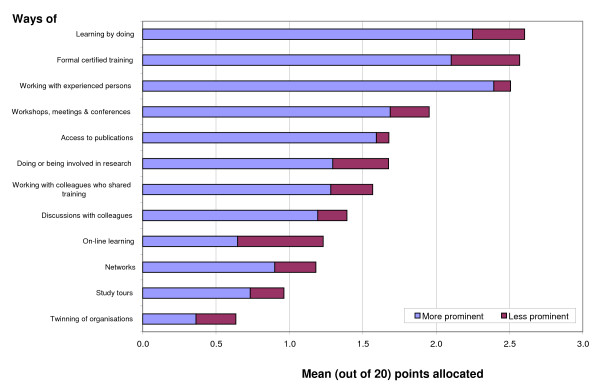
**Importance of ways of learning about health planning and management**.

A set of 12 questions asked respondents about the most important methods for their learning about planning and management of health services. Respondents were asked to allocate 20 points across 12 learning methods, allocating more points to the more important methods. In Fig. 1, the width of the bar (arithmetic mean number of points) represents the level of importance assigned by the participants to a learning method. The three methods reported as most useful were: "learning by doing", "formal certified training" and "working with experienced persons". Next most popular were "workshops, meetings and conferences", "access to publications" and "involvement in research". It was interesting to note that, contrary to some literature [23], our participants did not consider twinning and study tours as useful tools for capacity building, and did not place high values on networks or online learning.

Figure 1 also shows the percentage of respondents who thought the learning method should be more (blue section of the bar) or less (red) prominent in the health sector. The significance of the findings to this study is that the learning opportunities/training provided both at the Nuffield Centre and the NPMC included six of the top eight preferences (#3 is about the workplace and #6 is about research, not an objective of this course). The courses involved practical tasks on current issues (#1); carried formal certification (#2), even though they were not assessed; used a workshop format (#4); were supported with recent and relevant publications (some by the tutors) and bespoke materials (#5); and involved wide-ranging discussion with colleagues – some of whom were very experienced in some of the topics – and with tutors – who were experts in their particular fields (# 8). Finally, the participants were drawn from disparate places in terms of geography, role, responsibility and experience; one purpose of the training was to establish a community of practice and through this network enable participants to build on their shared training after the course (#7). While learning and application are not being measured directly here, the overlap between learning preferences and the learning opportunities provided bodes well for the significance of the courses in terms of potential application and organizational impact.

Generally, the results tallied with that of the first level of enquiry, i.e. the higher the usefulness of the learning methods to the respondent, the more the respondent thought it should be a prominent learning method. Overall, they thought most of the methods should be more prominently used, except for online learning and organizational twinning.

### Participants' satisfaction with learning techniques

Respondents were asked to identify which of 15 learning techniques had led them to achieve their current level of capability in their job. Then they rated their satisfaction with each technique on a four-point scale (not at all satisfied, a bit, mostly and very satisfied). Finally, respondents rated how important each method should be as a means for learning on a similar four-point scale, irrespective of how they had responded in the first or second stage.

Findings suggest that all 15 learning techniques listed in the questionnaire contributed, to varying degrees, in building individual capability. More than 90% mentioned reading textbooks and journals on health planning and management, use of online resources, participation in health management-related conferences/discussions, being part of a team responsible for planning or managing specific activities and attending in-service training courses for professional development. More than 80% mentioned taking advice from colleagues and doing action research in health planning and management. Respondents had the least experience with a job appraisal system (48%) and having a supervisor or mentor for developing new skills (57%).

The frequency of use of learning techniques did not overlap exactly with satisfaction levels. Respondents' satisfaction levels were highest with using on-line resources (although, as noted previously in Fig. 1, the respondents did not think this method should become more prominent), having a supervisor or mentor, attending in-service and professional development courses and doing action research. Respondents were least satisfied with taking advice from colleagues outside their place of work, using university libraries or materials sent from other organizations, and with their job appraisal system – all of which were less frequently used.

In terms of how important respondents thought these activities should be, the highest importance was placed on being part of a team that is responsible for planning and managing activities; doing action research; participating in conferences and discussions; and having a supervisor or mentor – all of which are workplace-based activities. The least importance was placed on taking advice from colleagues outside their place of work and using materials sent by other organizations. This latter finding echoes the low value given in Fig. 1 to the value of networking and organizational twinning.

### Overall views on the training courses

The respondents were asked their overall views on the short courses they had taken (Table 5). Respondents were asked to indicate their agreement with 15 statements on a five-point Likert scale. Some items were reverse-worded, where disagreement with the statement represents a favourable view towards the course.

**Table 5:** Views on the training courses

**Statement**	**View (n = 23)**		
	
	**Agree** **%**	**Neutral** **%**	**Disagree** **%**
A strength of this course is that it gave me the chance to meet other colleagues from different parts of the country.	95.5	5.5	0.0

The course made me realise the importance of continuous learning.	87.0	4.3	8.7

The teachers on the courses had academic credibility.	82.6	17.4	0.0

The course was interesting.	82.6	8.7	8.7

The course was relevant to the work I am required to do.	78.3	21.7	0.0

The course has changed my way of thinking.	78.3	13.7	8.7

The courses provided a linkage between training of individuals and institutional strengthening, so that the two reinforce each other.	76.2	9.5	14.3

There should be more in-country courses of this nature.	73.9	13.0	13.0

The course will help my career.	69.6	26.1	4.3

The course was worth the time it took.	66.7	19.0	14.3

The course has changed my ways of doing things.	52.4	33.3	14.3

There was an acceptable blend of theory and practice in the course.	45.5	31.8	22.7

There was too much emphasis on theory.	50.0	22.7	27.3

My boss did not value this course.	30.4	4.3	65.2

The course was too demanding.	54.5	36.4	9.1

Respondents agreed most strongly with the statements that a strength of the course was that it gave them a chance to meet colleagues from other parts of Iran, and that it made them realize the importance of continuing learning. More than 80% found the course interesting, with credible teachers, and more than 75% noted that the course was relevant to their work and institution and had changed their way of thinking. Their least favourable comments had to do with the demands of the course and the blend of theory and practice. Two thirds of respondents thought the course was worth the time it took and believed their bosses valued the course.

### Usefulness and application of the training

**Figure 2:**
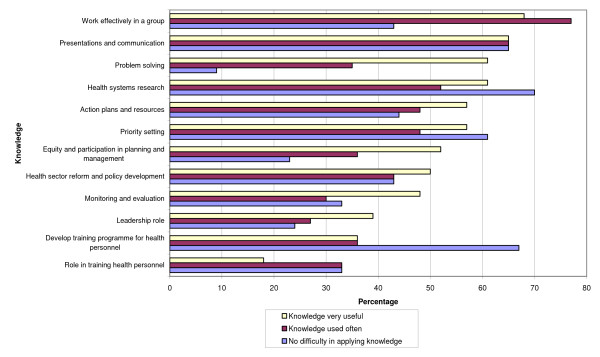
**Views on usefulness and application of knowledge from the training courses**.

Respondents were asked about the usefulness for their job of 12 areas of course-related knowledge, whether they used the knowledge often and how difficult it was to apply this knowledge in their work. Results are shown in Fig. 2.

The most useful areas of knowledge from the courses for their job were group work, making presentations, problem solving and health systems research. The least useful areas of knowledge, in their view, were roles in training health personnel, developing training programmes, and leadership roles. These results give an indication of participant satisfaction with training content. However, they are also important for another reason: the reported usefulness of an area of knowledge is a good predictor for its transfer to the job [24]. Figure 2 shows that this was generally true for the respondents, with the exception of four areas where they applied their new knowledge less often: problem solving, monitoring and evaluation, equity and participation in planning and management, and leadership roles. The respondents felt less difficulty in applying knowledge gained in the areas of health systems research, developing training programmes, making presentations and priority setting. This could be partly due to their background experience (74% had a background in policy, planning and management), as well as knowledge and skills acquired from the course (see below).

Figure 2 shows that the use of a knowledge area is generally lower when respondents find it difficult to apply in their job. Exceptions to this relationship include the areas of group work, problem solving, and equity and participation in planning and management. These areas were used more often, but the application of such knowledge was also considered difficult. Therefore, given the mixed results, it is difficult to generalize that there was a direct relation between the extent of use and the amount of difficulty in applying a particular area of knowledge in health planning and management.

On their return to work, 83% of the respondents said they were asked by their line managers about the course, indicating the organizational interest in the training, and 81% claimed they could perform their jobs better. The responses to an open-ended question on what new skills they learnt from the training can broadly be divided into two groups: one group subscribed to enhanced in-depth understanding of the health system, as indicated from a representative statement, "increasing knowledge about health sector reform in the field of planning, management, need assessment, priority intervention, and evaluation". The other group reported that their skills in communication and training were enhanced, as shown in this comment, "changing some of my teaching and assessment methods, doing evaluation for our planning in the department or faculty, focussing more on communication skills in training".

For the six respondents who thought the course had not helped them in performing their job, half attributed this to the course, saying that it was neither adequate nor well-planned. One participant said "It gave me knowledge, but it was not enough". A comment in a group discussion reinforced this: "we had problems... Health economics was not enough... Decentralisation, there is a gap between live theory and practice. The problem was on theoretical side. More topics could have been added to link with other areas of health management". However, the other three believed it was mainly due to the short time since they had returned to work, implying that they would be able to use their learning in the future.

## Discussion

### Evaluation methodology

The Kirkpatrick evaluation model proved useful for this project. Most training evaluations concentrate on the first two levels of participants' learning and reactions to the course. This evaluation emphasized assessing the application of training, since the course had key learning outcomes about improved functioning in management and training roles. Some information was also obtained about the impact on the organizations where participants worked, and on factors known to affect transfer of training.

There are a number of limitations to this evaluation. Because the participants attended different courses, and very few attended all the courses, they did not all evaluate precisely the same training experience. It is not possible to say whether some of the courses obtained very different evaluation results than others. The small size of the study population does not allow for statistically significant results. The short intervals between the conduct of the training and the evaluation for 10 of the respondents limited their ability to comment on the application of training. The questionnaires were long and the interviews time-consuming, and it would be necessary (and possible) to simplify the methods, for optimal use by the NPMC and the Nuffield Centre. The assessment of participants' learning did not distinguish between information/skills outcomes and affective outcomes (changes in attitude and motivation) as they were not explicit objectives of the course. However, the latter are important for renewing motivation and commitment of professionals and change agents [9]. When questions were asked about application of new knowledge and skills, the questions were simply about use/non-use, and not gradations of use, e.g. novice to expert level, which would help to assess the impact on the organization.

Published research concerning the impact of training university teachers is unanimous in its conclusion that there is little evidence available of impact at the level of application [25,26]. Two concerns outlined by the researchers are that: (1) much of the evidence of success that is claimed is based on self reporting by the participants; and (2) it is not possible to tell whether the training was the cause, or whether it was simply a case of accumulating greater experience of teaching (invariably, there are no control groups). Both of these concerns apply here. It is also difficult to assess to what extent the participants' responses were constrained by norms of courtesy to foreigners and respect for teachers, which were indicated to be strong values in Iran. Information was not obtained on the context of the respondents, which made it impossible to assess to what extent reported changes (or not) in practice were related to the training programme or to factors in the organizational environment, such as supportive supervision.

### Potential impact of the training

One of the purposes of the training programme was to enable participants to perform better in their health management roles, and participants reported that this was achieved overall. Many respondents noted the importance of training and development for learning new functions in health planning and management. As one respondent wrote: "The most important function is transferring the knowledge and skills to colleagues at provincial and district level, not only in their own provinces, but also in other provinces." This observation is commensurate with the lack of organized in-service training for health managers in the Iranian health system, although the capacity built at NPMC through this project will help to change that.

Short courses are considered effective for imparting information and for training in discrete skills [10]. The participants in these courses indicated that usefulness and application were highest in skills such as group work and making presentations, and in defined knowledge areas, such as health systems research and priority setting. Other areas such as leadership and training showed lower scores for usefulness and application. Given that one of the objectives was to build trainers' capacity at NPMC, the relatively lower scores given for training content were surprising, and would ideally have been followed up to obtain additional feedback.

The primary factors that influence transfer of training are learner characteristics, the design and delivery of the course and factors in the work environment [13]. In this first, experimental phase, some participants were selected partly on the basis of their English language ability, rather than on job function and potential, which may have reduced the impact of the training on their organizations. Since Farsi will be the medium of communication, language will not be an issue in future Iranian courses, although the appropriate selection of trainees will remain crucial to the success of courses offered by NPMC in terms of organizational impact. While the design and delivery of the courses was considered satisfactory overall, transfer of training to the job could have been enhanced by placing more explicit emphasis on learning outcomes detailing such application, with content including a requirement for participants to set their own short-term and long-term transfer goals [13].

Several factors in the work environment reduced application of learning. A number of interviewees indicated that they had not yet had the time or opportunity after the course to apply their new knowledge and skills. One said: "After the course, nobody asked us to do anything on clinical governance." Another noted that the "manager...does not allow or give opportunity to the people trained to do." Another problem they indicated was the changing context of health sector reform, so that people were reassigned to new priorities that did not necessarily require the knowledge and skills they had acquired from the courses.

One of the major factors in the work environment that limits the transfer of learning is the lack of involvement by senior management [6]. However, participants believed that their bosses valued the course and some were formally asked to train their colleagues. This makes it more likely that the bosses would be positive about the courses' potential applicability, and would support and the growth and development of trained staff.

### Training methods and content

The course was based on principles of adult learning, and this was reflected in the generally positive comments on satisfaction with and importance of the different training methods used. A problem noted with the course was the gap between theory and practice. It is difficult for trainers external to an organization (and the country), who do not know its context and operations intimately, to develop training content that directly addresses issues particular to an organization, even if they are working with a relatively detailed training needs assessment, as this project was. The external trainers had to rely mainly on discussions and exercises in which participants were asked to apply general principles to issues they identified in their own organization. Ideally, the external trainers would have worked with local managers and trainers to develop materials and content that was closely aligned with actual situations in the health system. As one informant noted: "If you could involve lecturers who are involved in practice – who work in the field – it is very important." This process would have a secondary effect of helping local trainers to improve their planning for and content of courses.

Two important methods for building capacity of individuals deserve special mention. Few respondents had experience with learning through a job appraisal system or having a supervisor or mentor, although those who did have such experience valued it highly. Supervisory support and encouragement for the application of new knowledge and skills, which can be reflected in a job appraisal, are reported to improve training transfer, but these are functions of managers that are beyond the remit of trainers [27]. Their lack of use in the health system may be partly due to lack of training of managers in these methods.

It is surprising that participants did not value online learning and twinning of institutions, which are favoured by some international agencies and institutions [28]. Among possible reasons for not showing interest in online learning could be that it requires good information technology and Internet connectivity, which are often not available in remote parts of the country. Experience also shows that in health planning and management training, participants learn from interacting with peers and sharing of experience, which is more challenging with online learning. As information from respondents was gathered by using a self-administered questionnaire, some could have had difficulty in understanding the term "twinning of institutions" for two reasons. First, English is not the respondents' first language, and second, they may not have had experience of twinning with another international institution, as Iran has remained relatively isolated internationally for a long time.

### Capacity building for management, training and evaluation

This project was permitted to conduct a more thorough evaluation than is usual for international short courses, and the experience proved useful in several ways. It produced information that was helpful for NPMC and Nuffield Centre trainers in terms of participants' reactions to and learning from the course, as well as for the Ministry of Health and Medical Education in terms of application of learning. Trainers obtained ideas for improving future course content and methodologies and for evaluating courses.

As building the capacity of individuals alone has proved unsuccessful in many situations [29], the project involved aspects of institutional capacity building. This enabled the Ministry of Health and Medical Education to obtain ideas for improving capacity building through other methods besides training, such as mentoring and job appraisals, and feedback on the successful methods they already use, such as assigning employees to work with experienced persons. Testing and adaptation of innovations with visible successes create favourable conditions for institutional capacity building [1]. As strong and effective leadership for change from top management is an important success factor for institutional capacity building [1], data were produced for both the NPMC and the Ministry of Health and Medical Education that could justify funding future courses, including the need for advanced courses.

The process for developing the evaluation plan was unsatisfactory in terms of the professional development of the NPMC trainers. It could have been a part of a slightly longer training-of-trainers module: for example, planning for the evaluation, implementing it and analysing the results would have been an excellent practical assignment. The demand for the evaluation came from the trainers who attended courses at the Nuffield Centre, not from the Ministry of Health and Medical Education or NPMC, and the evaluation was developed and carried out by the Nuffield Centre with little input from the Iranian institutions. Of course, details of the evaluation results and methodology were made available to NPMC for adaptation to future programmes, and the value of the evaluation process was emphasized, but there was no opportunity to discuss the findings in detail and work with the two organizations to improve follow-up courses.

## Conclusion

The training programmes in Iran, and those that the Nuffield Centre might support in the future elsewhere, should continue to be characterized by principles of good practice: detailed training needs assessment, appropriate participant selection, learning outcomes, content and methods, and focused evaluation. Support from external trainers should focus on capacity development for all these principles.

Each course needs to prepare clear information about whom the course is aimed at, what the prerequisites are (academic or experience), and what the course will enable graduates to do, so that managers can select appropriate participants. In order to ensure a balance between theory and practice, trainers should collect local case studies and information and develop locally-oriented learning materials to illustrate theory. However, trainers also need to make explicit in the learning objectives that participants need to draw their learning not only from their country's specific situation but from other, external situations and apply what they have learnt into situations in the workplace [30]. Intramodular assignments could be better supported through distance learning methods, rather than through classroom discussion in succeeding modules with different teachers. For Iran and other countries, the courses should incorporate new learning objectives and content in areas identified as important: practical research, job appraisal, supervision and mentorship, and organizational change management processes.

The evaluation methodology that was developed for Iran provided useful guidance for trainers and is replicable, although with modifications needed to shorten and simplify it. The questions on training methods could be reduced (if training programmes continue to use the rated methods), and further questions added on motivation, level of application of learning, and the organizational factors affecting application.

Trainers should plan evaluation along with the courses, ensuring the use of the first two levels of evaluation for the majority of training. The third and fourth levels of evaluation could be implemented at appropriate points in the life of a training institution, such as when it is pilot-testing or consolidating training programmes, or carrying out strategic planning. The organizations sending trainees could be involved in planning of evaluation at these levels, given the importance of the information to them.

The training evaluation plans should be comprehensive, with clear objectives and users, diverse evaluation methods and tools, analysis plans that allow results to be triangulated and validated, and well-defined dissemination plans that can inform training activities, as well as human resource management and organizational development. Consistent use of evaluation over a few years should help to create demand for such information, and allow data to be compared from different training groups, so that trends can be analysed.

Those who fund training programmes should consider the capacity of local training staff to assess training needs, plan courses, evaluate them and apply the results for improvement. These aspects need to be built into contracts with external assistance, by incorporating a specific responsibility for capacity development, and allowing time for local and external trainers to co-develop and co-evaluate courses.

In Iran, as in other countries, it will be important to continue to carry out health management training, and to justify the large investments in training programmes, through rigorous assessment of their contribution to the capacity development of individuals, organizations and health systems.

## Competing interests

The Leeds authors, namely Drs Omar, Gerein, Pearson and Mr Butcher work for the institution that undertook to develop and conduct training both in Leeds and in Tabriz. Dr Tarin works for the organisation that funded the training programmes and Dr Heidari works for the institution that received training.

## Authors' contributions

MO made a substantial contribution to the conception and design, acquisition, analysis and interpretation of data. He was also involved in drafting the manuscript and revising it critically for important intellectual content. NG made a substantial contribution to the conception and design, analysis and interpretation of data. She was also involved in drafting the manuscript and revising it critically for important intellectual content. ET made a substantial contribution to the analysis and interpretation of data. He was also involved in drafting the manuscript and participated in revising it critically for important intellectual content. CB made a substantial contribution to conception and design of the study. He was also involved in revising and writing some sections of the manuscript. SP made a substantial contribution to the quantitative data analysis and producing required figures and tables. He was also involved reading and revising some sections of the manuscript. GH contributed to the conception of the study and writing the background section of the Iranian context. He was involved in reading and commenting on different drafts of the manuscripts. All authors have given final approval of the version to be published.

## Authors' information

MO is a senior lecturer in health organization and management at the Nuffield Centre for International Health and Development and a senior international health consultant with 30 years of developing country experience. His skills and interest are in the areas of policy analysis, decentralization and human resources development within the context of health systems development.

NG is a senior lecturer in sexual and reproductive health and monitoring and evaluation and is Head of the Nuffield Centre for International Health and Development. Areas of interest include reproductive health, health policy and systems, monitoring and evaluation and nongovernmental organizations. She has worked in Asian and African community health, planning and health sector reform.

ET is a health policy and systems specialist working with World Health Organization. He has extensive experience of public sector health management in Pakistan. He undertook his doctorate from Nuffield Centre for International Health and Development at the University of Leeds, United Kingdom. His research centred on "health sector reforms: factors influencing the policy process for government initiatives in the Punjab (Pakistan) health sector".

CB is the principal academic staff development officer at the University of Leeds. His areas of responsibilities include the University of Leeds Teaching Award (Ulta2), the Postgraduate Certificate in Learning and Teaching in Higher Education, the Postgraduate Diploma in Learning and Teaching in Higher Education, departmental development for learning and teaching, and the University Teaching Fellowship Scheme.

SP is a senior research fellow in reproductive health. He specializes in qualitative and quantitative research on men's and young people's reproductive health. Before joining the University of Leeds, he worked as a Teaching Fellow in the Department of Social Statistics at Southampton and as a Lecturer at the Centre for Population Studies, University of Zimbabwe. There he was involved in several research projects on maternity care, men's reproductive health and adolescents' reproductive behaviour.

GH is senior advisor to the Minister of Health and Medical Education of the Islamic Republic of Iran. He is responsible for the development and strengthening of the primary health care network in the context of health sector decentralization.
